# Evaluation of Cocktails with Recombinant Proteins of *Mycobacterium bovis* for a Specific Diagnosis of Bovine Tuberculosis

**DOI:** 10.1155/2014/140829

**Published:** 2014-07-08

**Authors:** María Laura Mon, Roberto Damián Moyano, Mariana Noelia Viale, María Alejandra Colombatti Olivieri, Ignacio José Gamietea, Valeria Noely Montenegro, Bernardo Alonso, María de la Paz Santangelo, Mahavir Singh, Rosario Duran, María Isabel Romano

**Affiliations:** ^1^Instituto de Biotecnología, INTA, Los Reseros y Nicolás Repetto, 1686 Hurlingham, Buenos Aires, Argentina; ^2^INTA, Estación Experimental Agropecuaria Delta, 1670 Delta, Buenos Aires, Argentina; ^3^SENASA, Dirección de Laboratorio y Control Técnico (DILAB), 1640 Martínez, Buenos Aires, Argentina; ^4^LIONEX Diagnostics & Therapeutics GmbH, 38126 Braunschweig, Germany; ^5^Unidad de Bioquímica y Proteómica Analítica, Institut Pasteur de Montevideo/Instituto de Investigaciones Biológicas Clemente Estable, 11400 Montevideo, Uruguay

## Abstract

The Delayed type hypersensitivity skin test (DTH) and interferon-gamma assay are used for the diagnosis of bovine tuberculosis (TBB). The specificity of these diagnoses, however, is compromised because both are based on the response against purified protein derivative of* Mycobacterium bovis *(PPD-B). In this study, we assessed the potential of two cocktails containing* M. bovis *recombinant proteins: cocktail 1 (C1): ESAT-6, CFP-10 and MPB83 and cocktail 2 (C2): ESAT-6, CFP-10, MPB83, HspX, TB10.3, and MPB70. C1, C2, and PPD-B showed similar response by DTH in* M. bovis*-sensitized guinea pigs. Importantly, C1 induced a lower response than PPD-B in* M. avium*-sensitized guinea pigs. In cattle, C1 displayed better performance than PPD-B and C2; indeed, C1 showed the least detection of animals either vaccinated or Map-infected. To optimize the composition of the cocktails, we obtained protein fractions from PPD-B and tested their immunogenicity in experimentally* M. bovis*-infected cattle. In one highly reactive fraction, seven proteins were identified. The inclusion of FixB in C1 enhanced the recognition of* M. bovis*-infected cattle without compromising specificity. Our data provide a promising basis for the future development of a cocktail for TBB detection without interference by the presence of sensitized or infected animals with other mycobacteria.

## 1. Introduction

The infection and disease produced by* Mycobacterium bovis*, which is the causative agent of bovine tuberculosis (TBB), is an important problem in cattle and other animal species. Human tuberculosis (TB) is produced mainly by* Mycobacterium tuberculosis*. However,* M. bovis* can also be responsible for the disease in humans, which makes this bacterium an important zoonotic species [1]. The TBB eradication programs in Argentina are based on a prompt detection of infected animals and their subsequent removal from the herd.

Delayed type hypersensitivity skin test (DTH), which is based on response against single intradermal inoculation of purified protein derivative of* M. bovis* (PPD-B), remains the primary surveillance tool to diagnose TBB in our country. The intensity of DTH reactions elicited in* M. bovis-*sensitized guinea pigs is the model to select batches of PPD-B to be used in the field. Thus, this animal model could be used to evaluate reagents with potential for TBB diagnosis. Disease control can also be facilitated by using the interferon-gamma (IFN-*γ*) assay. In their original form, these tests, DTH and IFN-*γ*, are based on the response against PPD-B. This reagent is a mix of proteins, lipids, and carbohydrates obtained from heat-killed cultures of* M. bovis* strain AN5, which compromise specificity because some of its antigenic components are present in others nonpathogenic and pathogenic mycobacteria [2, 3]. Animals infected with* Mycobacterium avium* subsp.* paratuberculosis* (Map), the etiologic agent of paratuberculosis (PTB), or vaccinated with BCG have demonstrated positive response in these assays using PPD-B. The specificity of these tests can be increased with the use of defined* M. bovis* antigens.

Many mycobacterial proteins have been isolated and evaluated in DTH and IFN-*γ* assays in animals with TBB [4–13]. For instance, ESAT-6 (Rv3875 or EsxA) is one of the major antigenic targets identified in both cattle and humans with tuberculosis [14]. Several other antigens with molecular masses of around 10 kDa and belonging to the Esx family of proteins have been identified subsequently, such as CFP10 (RV3874 or EsxB) and TB10.3 (Rv3019c or EsxR), and they have been shown to be equally well recognized by T cells [15]. ESAT-6 and CFP-10, or combinations of these antigens, have been extensively tested by DTH and IFN-*γ* assays in naturally* M. bovis-*infected cattle with satisfactory results [16–19]. The studies performed in* M. bovis-*infected cattle yielded promising results with cocktails containing ESAT-6, CFP-10 plus the addition of other antigens [18–20]. ESAT-6 and CFP-10 are encoded in a genomic region, RD1, that is absent in BCG Pasteur and, thus, are useful as diagnostic tools for differentiating TB-infected from BCG-vaccinated animals (DIVA) [19]. Proliferative immune response with synthetic peptides derived from the sequences of CFP-10 and ESAT-6 showed that BCG-vaccinated cattle did not respond to this peptide cocktail, whilst they all responded to PPD-B [21].

Furthermore, the use of a recombinant ESAT-6: CFP-10 fusion protein was useful to differentiate* M. bovis*-infected from those Map-infected cattle [22]. Several studies have identified immunogenic proteins present in both* M. bovis/M. tuberculosis* [4, 9, 10, 15, 17]. A disadvantage of those studies, however, is that they are restricted to a relatively small set of proteins biased by the initial selection criteria. Recently, a nonbiased approach with an extensive library of clones expressing mycobacterial proteins was used and 33 gene products that induced IFN-*γ* production in whole-blood cultures from TB-reactor animals were detected. One of these proteins did not induce responses in BCG-vaccinated animals, which is suitable for DIVA diagnostic. Greater responder frequencies were observed for BCG-vaccinated cattle than for TB reactors with the remaining antigens, which leads to speculate that other “less immunodominant” antigens maybe recognized to a greater extent in BCG-vaccinated animals [23].

These recent studies show that the search of other antigens for a more sensitive and specific diagnosis of TB is still relevant. Unfortunately, few studies have shown the feasibility of* M. bovis* antigens to increase specificity of DTH using either the guinea pig model or experimentally* M. bovis*-infected cattle [24–26]. Moreover, even more information of the use of cocktails with* M. bovis* antigens in naturally infected cattle remains scarce.

The present study aimed to assess cocktails containing previously defined antigens of* M. bovis* in order to differentiate animals with TBB from vaccinated- or Map-infected animals. For this purpose, we used different approaches: DTH in the guinea pig model and both DTH and IFN-*γ* assays in the cattle model. The specificity of the defined antigens was evaluated in both BCG-vaccinated and Map-infected cattle. Finally, novel cocktails enriched with immunogenic proteins identified by mass spectrometry analysis of selected antigenic fractions of PPD-B were evaluated in TBB, PTB, and free herds.

## 2. Material and Methods

### 2.1. Antigens

In the first part of the present study, we assessed the application of antigens that have been extensively tested in TBB with satisfactory results. These antigens were used to produce two cocktails: cocktail 1 (C1) containing ESAT-6, CFP-10, and MPB83 and cocktail 2 (C2) with ESAT-6, CFP-10, MPB83, MPB70, TB10.3, and HspX. The potency of the cocktails was evaluated by DTH in* M. bovis, M. avium* sensitized-guinea pigs, and nonsensitized guinea pigs, as well as in experimentally* M. bovis*-infected cattle (DTH and INF-*γ*), BCG-vaccinated, naturally Map-infected cattle, and in a free TBB and Map herd by IFN-*γ*.

The purified recombinant proteins, ESAT-6, CFP-10, MPB70, MPB83, TB10.3, and HspX, were kindly provided by Professor Mahavir Singh (Lionex Diagnostics and Therapeutics GmbH, Germany). In all cases, the endotoxin content was <1.27 IU/mg.

In the second part, we characterized immunodominant antigens from the PPD-B. The coding sequences of CFP2, FixB, and PepA were amplified by PCR using the following primers: CFP2-fw 5′-cgcggatccatgaagatggtgaaatcga-3′(BamHI site) and CFP2-rev 5′-ataagctttcagttccctgcggcctgc-3′(HindIII site); FixB-fw  5′-cgggatccatggctgaagtactgg-3′(BamHI site) and FixB-rev 5′-taagcttctagcccttgcgggcc-3′ (HindIII site); PepA-fw 5′-aaggatccatgagcaattcgcgccg-3′(BamHI site) and PepA-rev 5′-ataagctttcaggccgggggtccct-3′(HindIII site).* M. bovis* DNA was employed as template for the amplifications. The amplification programme was as follows: an initial step of 95°C for 10 min, 35 cycles of 95°C for 1 min, 58°C for 1 min, and 72°C for 1 min followed by a final termination step of 72°C for 8 min. The complete open reading frames of CFP2 (507 pb), FixB (957 pb), and PepA (1068) were subsequently cloned into the expression vector pRSET-A (Invitrogen BV, Leek, The Netherlands). The derived constructions were transformed into* Escherichia coli* BL21 (pLys) competent cells. A 3-mL inoculum of* E. coli* BL21 harboring pRSET-A with the gene inserts was diluted 1/20 and grown to the mid-logarithmic growth phase (OD of 0.6) in LB-ampicillin for 16 h at 37°C. One mM concentration of isopropyl-D-thiogalactopyranoside (IPTG) (Merck, Germany) was added to induce recombinant gene expression over 3 h. Cells were then harvested by centrifugation and frozen at −20°C until further use. The cell pellet was lysed with 1 mL of lysis buffer (Tris-HCl 100 mM pH 7.5, NaCl 300 mM, Glycerol 20%, NP40 1%) using a Fast Prep FP120 40 seg. 5.5 m/s (Bio101-Savant, Holbrook, NY). The expression of the soluble antigens was confirmed by both Coomassie blue staining of polyacrylamide gels and Western blot analysis using Mouse anti-His antibody as primary antibody (GE Healthcare, UK) and anti-Mouse Alkaline phosphatase as secondary antibody (Sigma, Missouri, USA). The recombinant proteins in the* E. coli* cell extracts were purified by using a Ni-NTA resin (Niquel nitrilotriacetic acid) (Qiagen Corp., CA). For this purpose, the supernatant was incubated for 90 minutes at 4°C with the resin and immobilized in the column. The resin was then washed with lysis buffer and eluted with increasing concentrations of imidazole (250 mM, 500 mM, and 1 M) (Sigma, St. Louis, MO) in buffer (Tris-HCl 50 mM pH 7.5, NaCl 500 mM, Glycerol 20%, and NP40 1%). The presence and purification of the protein was confirmed by Coomasie blue and Western blot. The protein concentration was estimated with the Micro BSA Protein Assay kit (Pierce, USA).

PPD-B and PPD-A were obtained from the National Service of Agricultural and Food Health and Quality (SENASA, Buenos Aires, Argentina) with a protein concentration of 1 mg/mL and 0,5 mg/mL, respectively.

### 2.2. Gene Nomenclature and Identity of Antigens of C1 and C2

The* cfp-10* gene of* M. bovis* (Mb3904) has 100% identity to* cfp-10* of* M. tuberculosis* (Rv3874), which encodes a 10 kDa culture filtrate antigen CFP-10 or EsxB.

The* esat-6* gene of* M. bovis* (Mb3905) has 100% identity to* esat-6* of* M. tuberculosis* (Rv3875), which encodes an early secreted antigenic target 6 kDa, ESAT-6, or EsxA.

The genes* cfp-10* and* esat-6* are absent in the genome of* M. bovis* BCG and Map.

MPB83 and MPB70 are highly homologous proteins within* M. tuberculosis* complex members and the orthologous in different members of the complex are virtually identical. However, they are major antigens that are highly expressed by* M. bovis* and considerably less abundantly expressed by* M. tuberculosis.* The* mpb83* gene (Mb2898) and* mpb70* gene of* M. bovis* (Mb2900) have 100% identity with their orthologous genes in* M. tuberculosis* (Rv2873 and Rv2875, resp.).

The genes* mpb83*,* mpb70,* and* hspX* in* M. bovis* have 100% identity with these genes in* M. bovis* BCG and they are absent from Map's genome. The* hspX* gene of* M. bovis* (Mb2057c) has 100% identity with the gene (Rv2031c) that encodes the 16-kDa alpha crystalline (Acr) protein of* M. tuberculosis* or heat shock protein X.

The TB10.3 or* esxR* gene of* M. bovis* (Mb3045c) has 100% identity with the gene (Rv3019c) of* M. tuberculosis*, which encodes a secreted* esat-6* like protein.

The protein TB10.3 of* M. bovis* has 100% identity with this protein in* M. bovis* BCG and 80% with the protein of Map.

### 2.3. Animal Models

#### 2.3.1. DTH Skin Test in* M. avium* and* M. bovis*-Sensitized Guinea* Pigs*

Two groups of guinea pigs were sensitized, one with* M. bovis*, another with* M. avium,* by intramuscular inoculation of 0.5 mL of a sterile heat-killed suspension of* M. bovis* strain AN5 or heat-killed suspension of* M. avium* strain D4ER, respectively. A group of nonsensitized guinea pigs was used to evaluate the specificity of the reagents. The sensitized animals were prepared by clipping the hair from the entire abdominal and flank areas. Thirty days after injection, the guinea pigs were used for testing PPD-B, PPD-A, C1, C2, Esat-6/CFP-10, and buffer phosphate saline (PBS) as a negative control. On each guinea pig, six sites for injection of PPD-B and the cocktails were selected. Three sites on each side of the midline and spaced at a sufficient distance (2-3 cm aprox.) from each other to avoid overlapping of skin reactions. The induration diameter in guinea pig was read at 24 h, according to protocol used by the National Service of Agricultural and Food Health and Quality (SENASA), Buenos Aires, Argentina.

C1 and C2 cocktails were prepared in PBS at a final concentration of 1 *μ*g/mL of each antigen. The animals received intradermal injections with 0.2 mL of each cocktail or PPD-B and PPD-A at 1 *μ*g/mL.

#### 2.3.2. Cattle

*(1) BCG-Vaccinated and Experimentally M. bovis-Infected Cattle.* One group of five castrated male Holstein-Fresian calves of three to four months of age was inoculated subcutaneously in the side of the neck with 1 × 10^6^ colony forming units (CFU) of BCG Pasteur suspended in PBS. Another group of six animals was infected with a wild boar virulent strain* M. bovis* 04-303 by intratracheal instillation of 5 × 10^7^ CFU as described previously [27]. The strain* M. bovis* 04-303 is an isolate obtained from a wild boar with tuberculous lesions [28]. Briefly, this inoculation procedure was carried out by anaesthetizing the calves with xylazine HCl (Rompun, Bayer, Germany; 0.1 mg/kg) intravenously and then inserting an 80 cm endotracheal tube into the trachea. A cannula was inserted through the endotracheal tube. The 1.5 mL inoculum containing the* M. bovis* 04-303 was injected through the cannula and flushed out with sterile saline equal to 10 mL volume.

All the animals used in this study were DTH negative to both PPD-A and PPD-B at the beginning of the experiments. These experimentally infected animals were part of a trial that included evaluation of candidate TBB vaccines [27].

To confirm that the animals were successfully infected, the calves were euthanized 100 days after infection and then thin slices of lungs and lymph nodes of the head and pulmonary region were analyzed in search of granuloma formations. All* M. bovis-*infected animals presented macroscopic lesions and were positive for both bacterial isolation and* IS6110*-PCR in both tissues [27].

Experimental vaccination and* M. bovis* infection were performed inside the biosafety BSL3 facilities for animals of the National Institute of Agricultural Technology (INTA), Argentina, in compliance with the regulations of the Ethical Committee of INTA (CICUAE) and the biosafety protocols as authorized by the National Service of Agricultural and Food Health and Quality (SENASA).

*(2) Naturally Map, M. bovis-Infected Cattle, and Free TBB and PTB Herd.* For the purpose of verifying the TBB cocktails specificity by IFN-*γ* assay, we used blood samples of 10 and 17 animals from 2 dairy herds with PTB confirmed by fecal culture. The selected animals were positive by DTH for PPD-A.

A total of 58 animals from* M. bovis*-infected beef herd were 27 DTH positive (induration reaction ≥5 mm), 19 displayed intermediate reaction (1–3 mm) and 12 animals were DTH negative.

As a negative control 10 animals from a free TBB and PTB herd were used for IFN-*γ* release assay.

### 2.4. Evaluation of C1 and C2 in BCG-Vaccinated, Experimentally* M. bovis*-Infected and Naturally Map-Infected Cattle by IFN-*γ* Release Assay

Blood samples from infected and vaccinated cattle were collected from the jugular vein into heparinized vials at 20, 30, 60, and 90 days after* M. bovis* infection (dpi) and 30 and 60 days after BCG vaccination (dpv). These samples were used to evaluate cocktails and fractions from PPD-B by IFN-*γ* release assay using a commercial ELISA-based kit (Bovigam; Prionics, Shlieren, Zurich, Switzerland). Briefly, aliquots of 200 *μ*L of blood were added in a 96-well culture microplate and incubated with 25 *μ*L of antigenic preparations per duplicate. Each of the constituent proteins was added to the cocktails at a concentration of 55 *μ*g/mL and the PPD-B peptidic fractions were used at a concentration of 36 *μ*g/mL of total protein. PPD-A and PPD-B were used at a final concentration of 50 *μ*g/mL. Negative-control wells with PBS alone were included for each tested animal. As positive control, 4.5 *μ*g/mL pokeweed mitogen (Sigma-Aldrich, United Kingdom) was used. The plates were incubated in a humidified 5% CO_2_ incubator at 37°C for 16 h. Stimulated plasma was obtained by centrifugation and IFN-*γ* concentrations in plasma were determined following the manufacturer's procedures. Color development was measured at 450 nm and the results were expressed as optical density (OD) indices (ODIs) (ODI = OD for antigens stimulated cultures/OD for PBS stimulated cultures). An ODI equals or higher than 2 was considered positive. The coefficient of variation between duplicate wells was less than 5%, and the OD for the control wells was usually less than 0.1.

Also, blood samples of 17 animals from a dairy herd with PTB, positive to DTH with PPD-A, were stimulated with C1, C2, PPD-A, or PPD-B to test specificity in the IFN-*γ* release assay.

### 2.5. DTH Skin Test in Experimentally* M. bovis*-Infected Cattle

The experimentally* M. bovis-*infected animals were tested by DTH before and after infection. Before infection, all the animals used in this study were DTH negative using PPD-B (<1 mm). The cattle were reevaluated by DTH 90 days after* M. bovis* challenge, using C1, C2, and PPD-B. The cocktails for DTH were prepared with 10 *μ*g of each antigen per 0.1 mL in PBS and 100 *μ*g per 0.1 mL of PPD-B. Animals were intradermally injected with 0.1 mL of C1, C2, or PPD-B (1 mg/mL) and the thickness of the caudal fold tuberculin skin test was measured using callipers before and 72 h after injection.

### 2.6. Antigen Identification in PPD-B Fractions

PPD-B was prepared and fractionated in order to identify new antigens components of the PPD-B capable to improve the immunological cellular response in animals with TBB. Briefly,* M. bovis* strain AN5 was cultured in modified Dorset-Henley medium for 8 to 10 weeks. The cultures were inactivated by autoclave at 100°C for 3 hours and the proteins were concentrated using a Pellicon XI device (Millipore Corporation, Bedford, MA) for tangential flow filtration.

#### 2.6.1. Separation of Protein Fractions from PPD-B

PPD-B proteins were divided into narrow molecular mass fractions by continuous elution of polyacrylamide gels as described previously [29]. Briefly, PPD-B preparation was resuspended in cracking buffer (SDS 2%, Tris HCl 0.125 M pH 6.8, 2-mercaptoethanol 1%, bromophenol blue 0.02%, and glycerol 10%), heated for 5 min in boiling water, and resolved by sodium dodecyl sulfate-polyacrylamide gel electrophoresis 12% (SDS-PAGE). Thirty fractions were collected using a whole-gel elutor (Bio-Rad Corporation). The protein concentration of the different fractions was estimated by the micro BSA Protein Assay kit (Pierce, USA). The fractions were dialyzed against PBS and kept frozen at −80°C until used.

#### 2.6.2. Identification of New Antigens from Bovine PPD Fractions by Mass Spectrometry

PPD-B fractions were evaluated for their capability to stimulate IFN*γ* release as previously described and the proteins present in the selected fractions with higher ODIs were identified by mass spectrometry. The selected samples were digested with sequencing-grade trypsin (Promega) by incubation at 35°C overnight. Prior to MS analyses, the digested samples were desalted using C18 reverse phase microcolumns (Omix Tips, Varian). Briefly, columns were preequilibrated with 20 *μ*L of an aqueous solution of 0.1% trifluoroacetic acid (TFA). After sample loading, microcolumns were washed with 0.1% TFA and eluted with 0.1% TFA in 60% acetonitrile.

The samples were evaporated in the speed vac, resuspended in 12 *μ*L of 0.05% formic acid and then injected in a nanoLC equipment (Proxeon easynLC, Thermo Scientific). Peptide separation was performed in a reverse-phase column (easy C18 column, 3 *μ*m; 75 *μ*m ID × 10 cm; Proxeon, Thermo Scientific) and peptides were eluted using a 0.1% (v/v) formic acid in water 0.1% (v/v) formic acid in acetonitrile gradient (0–60% acetonitrile in 60 min; flow 400 nl/min). Online MS detection/analysis was carried out in a linear ion trap mass spectrometer (LTQ Velos, Thermo Fisher Scientific Corp., USA) with nanospray ionization. Proteins were identified by NCBInr database searching (November 2009) with peptide* m/z* values using the MASCOT search engine (version 2.3.02) in the MS/MS ion search mode and the following search parameters: taxonomy* Mycobacteriun tuberculosis* complex; peptide tolerance 1.5 Da; fragment mass tolerance 0.8 Da; and methionine oxidation as the allowed variable modification. Significant protein scores and individual ion score (*P* < 0.05) were used as criteria for positive protein identification.

#### 2.6.3. Evaluation of Novel Enriched C1 by IFN-*γ*

The performance of the enriched C1 with the recombinant antigens identified from highly reactive fractions of PPD-B (novel cocktails C1 + FixB, C1 + CFP2) was assessed using blood from a beef herd with high prevalence of TBB (*n*: 58) by IFN-*γ* release assay. In addition, we also used blood samples from a dairy herd with PTB (*n*: 10) and from a TBB and PTB free herds (*n*: 10).

## 3. Results

### 3.1. Evaluation of the Biological Potency of C1 and C2 in Guinea* Pigs*

**Figure 1:**
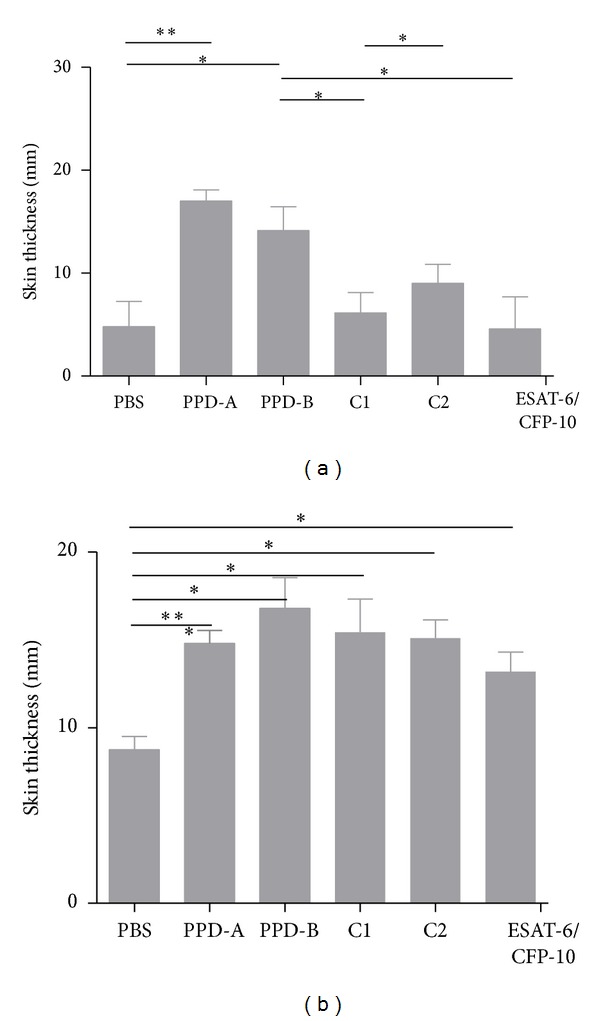
DTH response induced by PPD-A, PPD-B, C1, C2, ESAT-6/CFP-10, and PBS in guinea pigs previously sensitized with* M. avium* (a) or* M. bovis* (b). Responses were measured at 24 hs. Statistical differences between responses were found by using Kruskall Wallis test. (**P < 0.01; *P < 0.05).

We initially assessed the application of antigens that have been extensively tested in TBB with satisfactory results. These antigens were used to produce two cocktails: cocktail 1 (C1) containing ESAT-6, CFP-10, and MPB83 and cocktail 2 (C2) with ESAT-6, CFP-10, MPB83, MPB70, TB10.3, and HspX. The C1 and C2 were first tested in* M. bovis-* and* M. avium-*sensitized guinea pigs by DTH reaction by detection of swelling 24 hs after inoculation at the site of injection. The reactions in* M. bovis-*sensitized guinea pigs were similar to PPD-B when C1 or C2 was injected (Figure 1(b)). However, in* M. avium-*sensitized guinea pigs PPD-B had higher values than C1 (Figure 1(a)). Then C1 is a potential specific reactive for diagnosis of TBB (Figure 1). In order to evaluate if the addition of MPB83 to ESAT-6/CFP-10 compromises the specificity of C1 we tested ESAT-6/CFP-10 without MPB83. The response was not significantly different in* M. avium-*sensitized guinea pigs, and then we could infer that the addition of MPB83 to C1 would not compromise differential diagnosis.

When all the antigens were assayed in a group of nonsensitized guinea pigs, no reaction was detected (data no shown).

### 3.2. C1 and C2 Evaluation by IFN-*γ* Release in Experimentally* M. bovis*-Infected, BCG-Vaccinated, Naturally and Map-Infected Cattle

**Figure 2:**
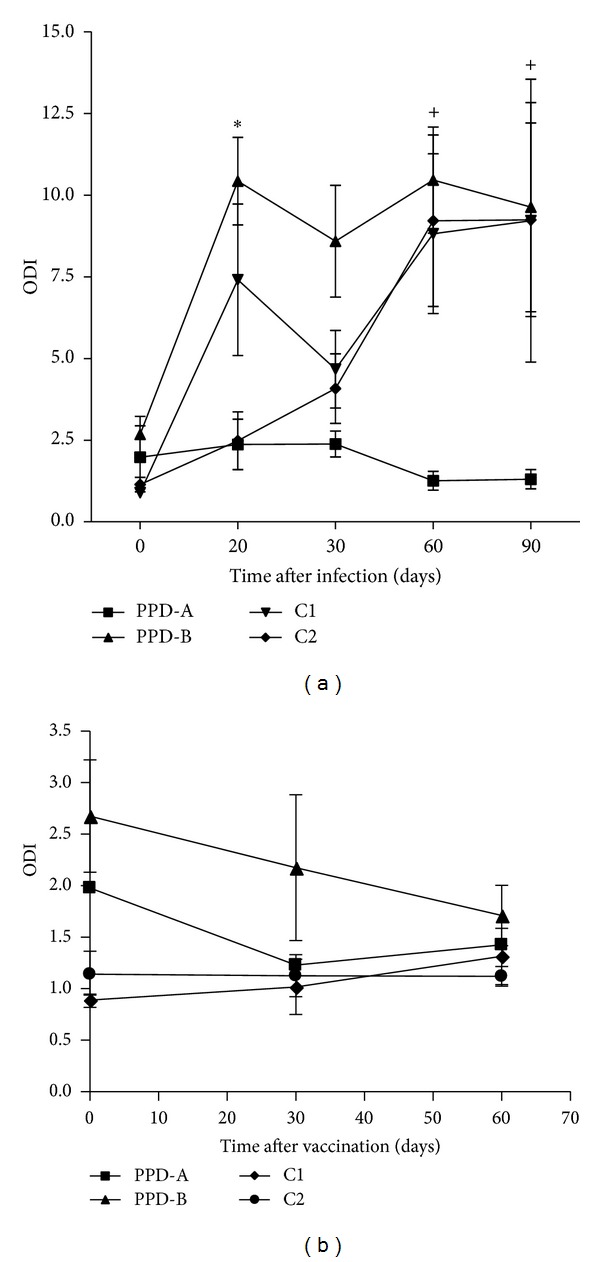
IFN-*γ* responses induced by PPD-A, PPD-B, C1, and C2 in experimentally* M. bovis-*infected cattle (*n* = 6) (a) and BCG-vaccinated animals (*n* = 5) (b) at different times. The cocktails were tested at a concentration of 55 *μ*g/mL per constitutive protein, PPD-A and PPD-B with concentration of 50 *μ*g/mL. Aliquots of 25 *μ*L of each antigenic preparation were added to 200 *μ*L of blood samples. The results are expressed as mean of ODIs with standard errors. Statistical differences between responses were found by using Mann Whitney test. *: differs (*P* < 0.05) from PDD-A and C2 values at 20 dpi for both PPD-B and C1.** +**: differs (*P* < 0.05) from PPD-A values at 60 and 90 dpi for PPD-B, C1, and C2.

The relative amounts of IFN-*γ* were measured stimulating blood from experimentally* M. bovis-*infected bovines using C1, C2, PPD-A, and PPD-B. No significant differences were found at 30, 60, and 90 dpi between C1, C2, and PPD-B. However, C1 and PPD-B stimulated higher values of IFN-*γ* than C2 20 dpi (Figure 2(a)). In BCG-vaccinated animals (*t* = 30 and 60 dpv), no significant differences were detected between the three antigens. However, prior vaccination (*t* = 0), IFN-*γ* levels were above 2 when blood was stimulated with PPDB (Figure 2(b)). For the purposes of this study, an ODI equals or higher than 2 was considered positive.

**Figure 3:**
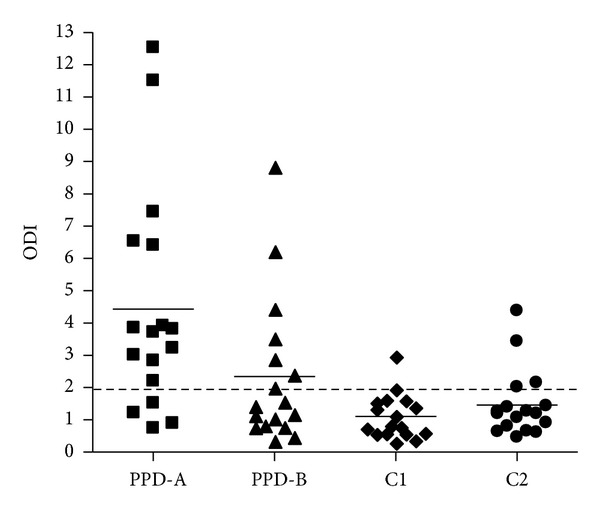
IFN-*γ* responses induced by PPD-A, PPD-B, C1, and C2 in naturally Map-infected cattle (*n* = 17). The cocktails were tested at a concentration of 55 *μ*g/mL per constitutive protein. PPD-A and PPD-B were assessed with concentration of 50 *μ*g/mL. Aliquots of 25 *μ*L of each antigenic preparation were added to 200 *μ*L of blood samples. The results for each animal are represented by different figures and the horizontal line provides the mean of the ODIs. The dashed line represents the cutoff values used for positivity.

Regarding the relative amounts of IFN-*γ* induced by C1, C2, and PPD-B in naturally Map-infected cattle, PPD-B and C2 detected 6/17 and 4/17 positive animals, respectively, while C1 detected only one (Figure 3). These results demonstrated that C1 is more specific for TBB diagnosis than C2 and PPDB, since this cocktail was effective in suppressing the detection of animals infected with other mycobacteria.

### 3.3. C1 and C2 Evaluation by DTH in Experimentally* M. bovis*-Infected Cattle

**Figure 4:**
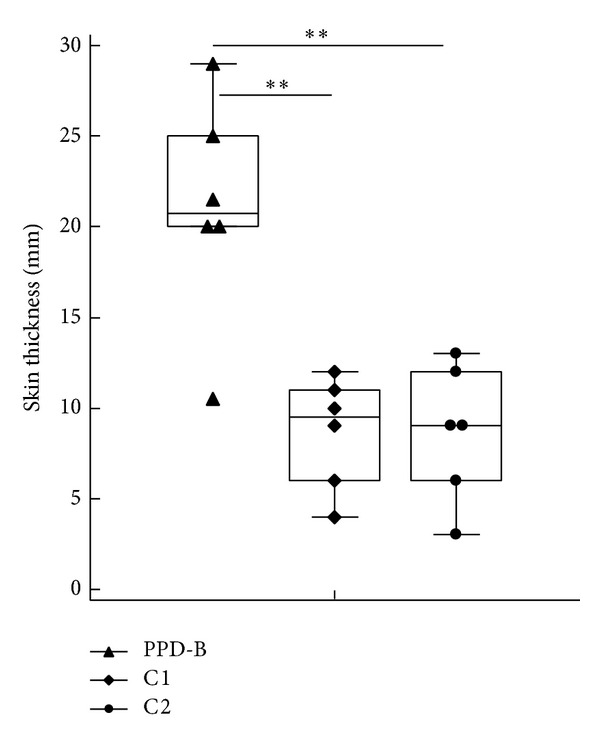
DTH response induced by PPD-B, C1, and C2 in experimentally* M. bovis-*infected cattle to 90 dpi (*n* = 6). The cocktails were prepared with 10 *μ*g of each antigen per 0.1 mL in PBS and PPD-B 100 *μ*g per 0.1 mL. The animals were intradermally injected with 0.1 mL of C1, C2, or PPD-B (1 mg/mL) and the thickness of the caudal fold tuberculin skin test was measured using callipers prior and 72 h after injection. Animal responses were represented by boxes and the horizontal line provides the median with standard errors. The statistical difference between responses was determined by using Kruskall Wallis test (**P < 0.01).

*M. bovis*-infected cattle were tested for DTH 90 dpi, using C1, C2, and PPDB as antigens. Values at the induration area were significantly lower when injecting C1 and C2 than swelling at the site of injection of PPD-B. However, if skin test is considered as positive (values above 5 mm), C1 and C2 detected 5/6 reactors to PPD-B in* M. bovis*-infected animals (Figure 4).

It is worth to mention that C1 and C2 were intradermally injected at a concentration of 10 *μ*g of each antigen per 0.1 mL in PBS, while PPD-B was inoculated at 100 *μ*g/0.1 mL. This higher concentration could be responsible for the differences to the reactions in response to PPDB.

### 3.4. Evaluation of PPD-B Fractions by IFN-*γ* in Experimentally* M. bovis*-Infected and BCG-Vaccinated Animals

**Figure 5:**
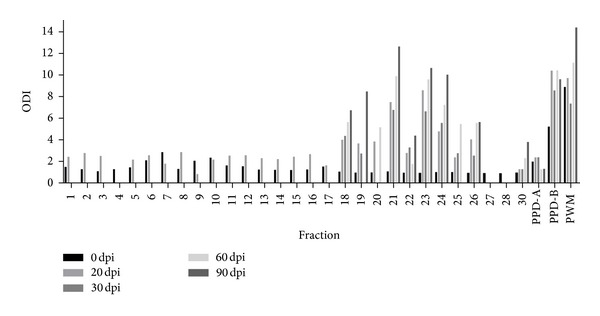
IFN-*γ* responses induced by PPD-B fractions in experimentally* M. bovis-*infected cattle (*n* = 6). PPD-B fractions were tested at a concentration of 36 *μ*g/mL. Aliquots of 25 *μ*L of each fraction were added to 200 *μ*L of blood samples. The results for each animal are represented by different vertical lines. The fractions were first tested with blood of animals from 0 and 20 dpi. The fractions that displayed the more stimulant responses were tested again at 30, 60, and 90 dpi.

**Figure 6:**
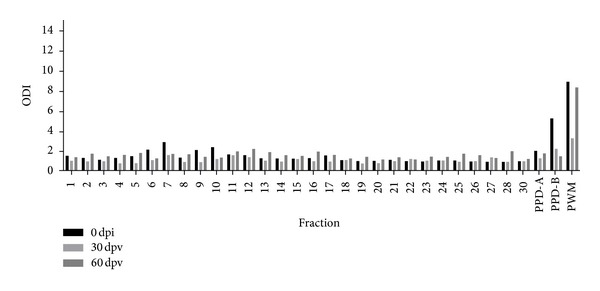
IFN-*γ* responses induced by PPD-B fractions in BCG-vaccinated cattle (*n* = 5). PPD-B fractions were tested at a concentration of 36 *μ*g/mL. Aliquots of 25 *μ*L of each fraction were added to 200 *μ*L of blood samples. The results for each animal are represented by different vertical lines. The fractions were tested with blood of animals taken at 0, 30, and 60 dpv.

Thirty protein fractions with molecular masses ranging from <17 to 90 kDa were obtained by electroelution of PPD-B in a 12% SDS-PAGE. These fractions were used to stimulate blood from experimentally* M. bovis-*infected and BCG-vaccinated cattle by IFN-*γ* release assays. All fractions were tested with blood taken at 0 and 20 dpi. We observed that PPD-B fractions with the lowest molecular masses were more antigenic. Therefore, these samples were further checked with blood taken at 30, 60, and 90 dpi. (Figure 5). These samples were also used to test blood from BCG-vaccinated cattle by IFN-*γ* release tests. Blood was collected at 30 and 60 dpv and most PPD-B fractions showed low levels of ODI (Figure 6). The fractions 21, 23, and 24 were selected for protein identification.

### 3.5. Protein Identification from Selected PPD-B Fractions by Mass Spectrometry

Selected fractions were analyzed by mass spectrometry to identify individual proteins. The three fractions were subjected to the LC-MS/MS but only one fraction (number 23) allowed the identification of seven putative antigens: CFP10, CFP2, MPB70, MPB83 FixB, PepA, HspX, and a partial sequence of an unknown protein (Table 1). CFP10, MPB70, MPB83, and HspX are known as antigenic proteins and were already included in C1 or C2. CFP2 belongs to the CFP protein family which is the founding member of the family; CFP10 is a putative secreted protein that may play a role in the development of protective immune response. FixB is an electron transfer flavoprotein that functions as a specific electron acceptor for other dehydrogenases. PepA is a probable serine protease. Finally, the detected partial sequence yielded significant alignments with a possible transport protein, SecE2 (*M. tuberculosis* Rv0379), with a sequence identity of 60% (70/116 aa).

### 3.6. Evaluation of the Novel Recombinant Mycobacterial Proteins from PPD-B Fractions

The genomic sequences corresponding to* cfp2, fixb, and pepa* were cloned, expressed, and purified as described above. We first checked the specificity of the antigens by IFN-*γ* in Map-infected animals. CFP2 and FixB did not detect any animals with PTB, while PepA detected 2 animals in a herd with PTB (data not shown).

Based on our results, C1 displayed better performance diagnostic than PPD-B and even better than C2 by IFN-*γ* assay; indeed, C1 detected animals with TBB and showed the least detection of animals either vaccinated or infected with Map. Thus, we prompted to improve C1 with the addition of the recombinant proteins, CFP2 or FixB.

**Figure 7:**
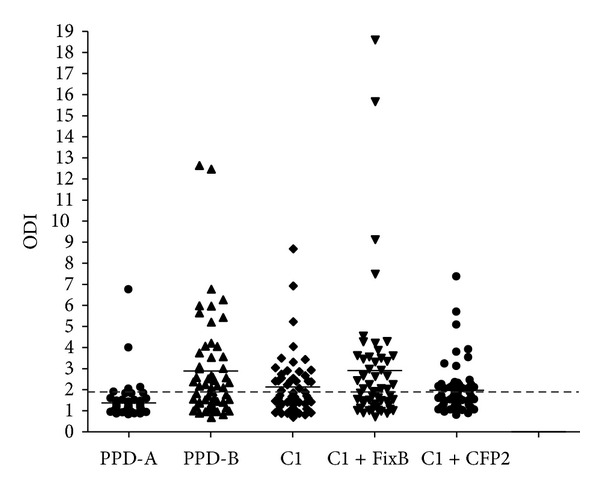
IFN-*γ* responses induced by PPD-A, PPD-B, C1, C1 plus FixB and C1 plus CFP2 in cattle from a herd with TBB (*n* = 58). The cocktails were tested at a concentration of 55 *μ*g/mL per constitutive protein, PPD-A and PPD-B with concentration of 50 *μ*g/mL. Aliquots of 25 *μ*L of each antigenic preparation were added to 200 *μ*L of blood samples. The results for each animal are represented by different markers and the horizontal line provides the mean of the ODIs. The dashed line represents the cutoff values used for positivity.

The novel cocktails were tested in 58 animals from a herd with TBB. According to the average value of IFN-*γ*, the response to PPD-B was similar to that of C1 with FixB (average ODI = 3) (Figure 7). PPD-B detected 33/58 animals, while C1 detected 24/58 animals suspected to be infected with* M. bovis*. The addition of FixB to C1 improved the results, detecting 29/58 animals. By contrast, the addition of CFP2 did not yield better results, detecting 22/58 (Figure 7, Table 2).

**Figure 8:**
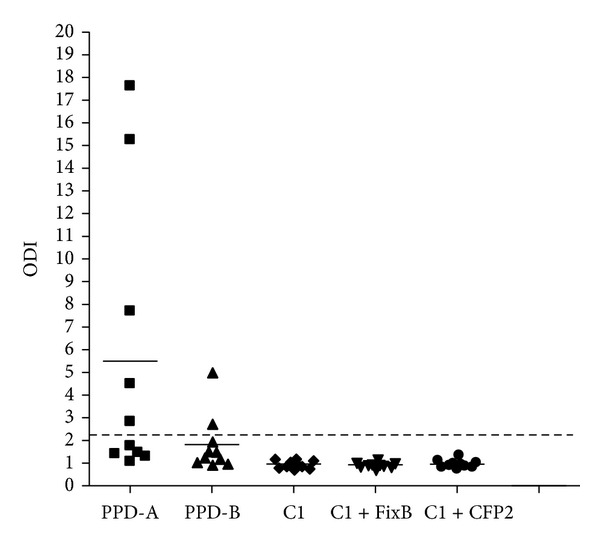
IFN-*γ* responses induced by PPD-A, PPD-B, C1, C1 plus FixB and C1 plus CFP2 in cattle from a herd with PTB (*n* = 10). The cocktails were tested at a concentration of 55 *μ*g/mL per constitutive protein, PPD-A and PPD-B with concentration of 50 *μ*g/mL. Aliquots of 25 *μ*L of each antigenic preparation were added to 200 *μ*L of blood samples. The results for each animal are represented by different figures and the horizontal line provides the mean of the ODIs. The dashed line represents the cutoff values used for positivity.

**Figure 9:**
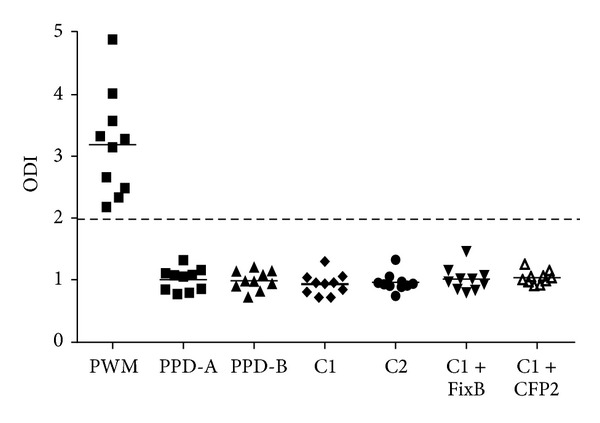
IFN-*γ* responses induced by PPD-A, PPD-B, C1, C2, C1 plus FixB and C1 plus CFP2 in cattle from a free TBB and PTB herd (*n* = 10). The cocktails were tested at a concentration of 55 *μ*g/mL per constitutive protein, PPD-A and PPD-B with concentration of 50 *μ*g/mL. Aliquots of 25 *μ*L of each antigenic preparation were added to 200 *μ*L of blood samples. The results for each animal are represented by different markers and the horizontal line provides the mean of the ODIs. The dashed line represents the cutoff values used for positivity.

Most importantly, C1, C1 plus FixB and C1 plus CFP2 did not detect animals neither in a PTB herd nor in healthy cattle (Figures 8 and 9). Thus, the inclusion of FixB in C1 enhanced the recognition of naturally* M. bovis*-infected cattle without compromising specificity.

## 4. Discussion

Several immunodominant proteins identified from* M. tuberculosis* and* M. bovis* have been identified by comparative genomics [4], differential transcription rates [5], or gene expression profiles associated with latent mycobacterial infection [6, 7]. Others have been detected from members of the PE/PPE family [8], proteins from crude protein fractions of* M. bovis,* and potentially secreted proteins. Within these potentially secreted proteins, we can mention members of the Esx family, such as ESAT-6, CFP-10, and TB10.3 (Rv3019c) [10, 11, 15].

CFP-10, ESAT-6, and TB10.3 are members of a large family of mycobacterial proteins, typically consisting of about 100 amino acids and are characterized by their organization in pairs within the genome [30]. Members of this family have been identified as potent T-cell antigens [15, 31]. Our group has previously participated in a multilaboratory study that assessed the sensitivity and specificity of the IFN-*γ* assay using several antigens in cattle naturally infected with* M. bovis* from Northern Ireland, Mexico, and Argentina. These regions have low, medium, and high prevalence of TBB, respectively. In the three countries, ESAT-6 and CFP-10 performed as superior diagnostic antigens [16]. Other previous studies have demonstrated that antigens of* M. bovis,* such as MPB70 and MPB83, also induced strong proliferation and IFN-*γ* responses in vitro in* M. bovis*-infected animals, while BCG-vaccinated or* M. avium*-sensitized animals did not respond to these antigens [19]. These results thus confirm that MPB83 and MPB70 also might be suitable antigens to differentiate between animals with TBB from animals with PTB- or BCG-vaccinated as well as from animals vaccinated against PTB. MPT83 (Rv2873) is a cell wall-associated lipoglycoprotein of* M. tuberculosis* whose function is still unknown. However, this protein has been suggested to play a role in adhesion and dissemination based on sequence analysis. Its homologue in* M. bovis*, MBP70, is also a serodominant antigen during* M. bovis* infection in cattle. MPB83 and MPB70 are major antigens highly expressed by* M. bovis* and considerably less abundantly expressed by* M. tuberculosis* [32–34].

In a prior study in our laboratory, eleven proteins were detected during the evaluation of the fractions from filtrate and cell extracts from* M. bovis* that elicited IFN-*γ* response in animals with TBB. Among the detected proteins, EsxI and HspX triggered a high T cell immune response as measured by IFN-*γ* release assay [9].

According to these previous results we selected the recombinant proteins included in each cocktail. Initially, cocktails composed of purified recombinant* M. bovis*/*M. tuberculosis* antigenic proteins HspX, TB10.3, ESAT-6, CFP-10, MPB70, and MPB83 were formulated. C1 contained the following proteins: ESAT-6, CFP-10, and MPB83 and C2 contained ESAT-6, CFP-10, MPB83, HspX, TB10.3, and MPB70.

When the potency of these cocktails was evaluated by DTH in* M. avium*-sensitized guinea pigs, C1 was more specific than PPD-B. C1 did not show significant differences in comparison with the response of CFP-10/ESAT-6; then in this experience the inclusion of MPB83 to C1 did not compromise differential diagnosis. However, one* M. avium*-sensitized animal showed response against protein cocktail that included ESAT-6 and CFP-10 antigens, absent in* M. avium*, but this response was similar to the controls inoculated with PBS.

In addition, when using protein-antigen combination of ESAT-6, CFP-10, and MPB83 in the IFN-*γ* released assay, C1 was highly sensitive and specific in the detection of experimentally* M. bovis-*infected cattle. This combination elicited no response in BCG-vaccinated calves and showed the least detection of Map-infected animals. On the other hand, C2 had poor IFN-inducing capacities in experimentally* M. bovis-*infected cattle at 20 dpi. Despite the immunogenicity of MPB70, Hspx, and TB10.3, their inclusion in C2 did not increase the IFN-*γ* response compared with the use of only ESAT-6, CFP-10, and MPB83 at 20, 30, 60, and 90 dpi. Since some of these antigens as ESAT-6 and CFP-10 are with TB10.3 members of the same family and MPB70 and MPB83 are closely related sharing 73% protein sequence identity, it is likely that their combination may result in antigen redundancy. C1 formulated with dominant T cell antigens, ESAT-6 and CFP-10, also contained MPB83. B antigens such as MPB70 and MPB83 may still have a role in promoting reaction initiation of DTH response, thereby, can help to elicit a better response to dominant effector antigens [13].

In the current study, 5/6 PPD-B reactors were also positive to DTH skin reactions when using C1 and C2 as immunogens in experimentally* M. bovis*-infected cattle. DTH response in experimentally* M. bovis*-infected cattle was significantly higher when injecting PPD-B than when using C1 or C2. This latter difference could be due to the lower concentration of each protein, 10 *μ*g in C1 and C2, compared to 100 *μ*g of PPD-B. In previous field studies, this concentration improved skin test responses without compromising specificity [13, 17]. The results of this study suggest that the doses of the recombinant proteins in the cocktails C1 and C2 for tuberculin skin test may have to be optimized.

The sensitivity of the DTH is less than 80%, which makes it unlikely as an only diagnostic tool for an efficient eradication of tuberculosis from a herd [35]. Therefore, in spite of the higher cost and complexity, the IFN-*γ* release assay can be used as a complementary test to the intradermal skin test, to confirm or discard the first results. The use of C1 in the IFN-*γ* release test demonstrated clear benefits since less responses were obtained, when compared with PPD-B, in BCG-vaccinated, or Map-infected calves; however, the results confirmed that the specificity of the test is compromised, when PPD-B is used as immunogen in cattle sensitized or infected with other mycobacteria. A minimum difference in the performance of C1 was observed between herds with PTB (Figures 3 and 8); it could be attributed to different environmental exposure. Finally, the addition of FixB to C1 resulted in higher sensitivity than C1, when evaluated in a herd with TBB. On the other hand, the addition of CFP2 to C1 did not result in a significant improvement in IFN-*γ* assay with C1.

Even though less animals were recognized with C1 plus FixB compared with PPD-B, the novel cocktail was more specific than PPD-B. Most important, in spite of the fact that only 58 bovine with TBB were tested, the inclusion of FixB in C1 enhanced the recognition of these naturally* M. bovis*-infected cattle without compromising specificity of IFN-*γ* assay, demonstrated that a C1 plus FixB could be suitable candidates for the development of diagnostic reagents to either differentiate between* M. bovis*-infected and MAP infected/BCG-vaccinated animal and then improve the specificity of the diagnosis of TBB.

## 5. Conclusions

This study demonstrates that cocktails containing defined* M. tuberculosis* complex antigens, such as ESAT-6, CFP-10, MPB83, and FixB can provide a sensitive and specific diagnosis of TBB. This finding is relevant, since a DIVA and a differential diagnosis between animals with TBB and PTB is needed. BCG vaccination could be applied in combination with such DIVA tests, together with PTB vaccines. These important proof-of-principle data provide a basis for future optimization and improvement of protein concentration in the novel cocktail.

## Figures and Tables

**Table 1:** Identified antigens from PPD-B by mass spectrometry.

Accession number	Rv	Mb	Proteins	Score	Length (aa)
gi 15611010	Rv3874	Mb3904	10 kda culture filtrate antigen EsxB or CFP10	182	100
gi 149926	Rv2875	Mb2900	MPB70	135	193
gi 2149409∗			Unknown	113	116
gi 15609513	Rv2376c	Mb2397c	Low molecular weight antigen CFP2	102	168
gi 15610165	Rv3028c	Mb3054c	Electron transfer flavoprotein subunit alpha FixB	85	318
gi 15607267	Rv0125	Mb0130	Serine protease PepA	70	355
gi 248681∗∗			MMP = 19 kDa major membrane protein [M. *tuberculosis* Erdman strain 107]	73	143
gi 6469702	Rv2873	Mb2898	MPB83 (Mycobacterium *tuberculosis*)	63	220

*Sequence producing significant alignments with the protein of *M. tuberculosis* Rv0379, possible protein transport protein SecE2, with identities in 70 from 116 aa.

∗∗Sequence producing alignments with HspX of *M. tuberculosis/M. bovis*.

**Table 2:** Number of recognized naturally *M. bovis* infected animals by the different antigens by IFN-*γ* release assay.

Antigen	Number of positive animals by IFN-*γ*/total
PPD-B	33/58
C1	24/58
C1 + FixB	29/58
C1 + CFP2	22/58
